# Supplementary material for: Addressing information and credit barriers to making India open defecation free and improving child health: Evidence from a cluster randomized trial in rural India
Source: PLoS One. 2025 Feb 27;20(2):e0318198. doi: 10.1371/journal.pone.0318198 (PMC11867337; doi:10.1371/journal.pone.0318198)
Supplement: S1 File — (DOCX) [file pone.0318198.s001.docx]

# Appendix

## Appendix 1: Evaluating the dissemination of BCC intervention

SI Table 1 reports that the intervention was successfully implemented as 98% of all the women respondents had participated in the behavior change communication (BCC) campaign and all 100% had heard of it in cluster A. Less than 2% of the respondents in cluster B and the control group had heard of the community-led total sanitation (CLTS) methodology, while none of them had participated in it. Our partnering organization, Grameen Development Services (GDS) was the primary source of carrying out the main messages in the CLTS module. More than 96% of the respondents in cluster A had participated in the core activities related to CLTS namely: OD mapping, fecal calculation, transect walk, calculating health expenses, fecal-oral demonstration, and arousing dignity [41]. 96% of the women in cluster A also participated in the handwashing training, implying the success of the intervention. The corresponding percentages for cluster B and the control group were zero, as the respondents had not participated in any CLTS intervention-related activities or handwashing training.

| SI Table 1: Intervention Check | | |
| --- | --- | --- |
|  | Sample Mean | Observations |
|  | (1) | (2) |
| Number of respondents | 339 |  |
| Number of villages | 15 |  |
|  |  |  |
| *Percentage of respondents who have heard of CLTS* |  |  |
| Cluster A | 100.00 | 339 |
| Cluster B | 1.11 | 3 |
| Control Group | 1.69 | 4 |
|  |  |  |
| *Sources of CLTS (percentage of respondents reported hearing CLTS through the following sources in cluster A)* |  |  |
| Newspaper | 0.00 | 0 |
| Television | 0.00 | 0 |
| Internet | 0.00 | 0 |
| Government-sponsored activities | 1.77 | 6 |
| GDS sponsored activities | 97.05 | 329 |
| Other NGO-sponsored activities | 0.00 | 0 |
| Other people living in the village | 2.95 | 10 |
| Other people living in other villages | 0.00 | 0 |
|  |  |  |
| % of respondents citing GDS as the most important source of hearing about CLTS (in cluster A) | 97.05 | 329 |
|  |  |  |
| *Percentage of respondents who participated in BCC* |  |  |
| Cluster A | 98.53 | 334 |
| Cluster B | 0.00 | 0 |
| Control Group | 0.00 | 0 |
|  |  |  |
| *% of respondents participated in following BCC activities (in cluster A)* |  |  |
| Fecal calculation | 96.76 | 328 |
| OD mapping | 97.64 | 331 |
| Transect walk | 96.76 | 328 |
| Calculating health expenses | 97.94 | 332 |
| ODF pledge/commitment | 88.50 | 300 |
| Handwashing training | 96.46 | 327 |
| Toilet maintenance training | 88.20 | 299 |
| Natural leader selection | 74.34 | 252 |
| Action plan | 75.81 | 257 |
| Fecal-oral demonstration | 97.94 | 332 |
| Follow-up monitoring | 89.97 | 305 |
| Arouse dignity (eating other's feces) | 97.64 | 331 |
| CLTS committee selection | 58.70 | 199 |
|  |  |  |
| *% of respondents stating the 3 most recalled activities in CLTS* |  |  |
| Fecal-oral demonstration | 43.95 | 149 |
| Arousing dignity (realizing that the community is eating each other's feces) | 24.19 | 82 |
| Follow-up Monitoring | 10.32 | 35 |
|  |  |  |
| *Notes*: Column (1) shows sample means of the responses obtained post-intervention in the project villages. One index woman from each household was interviewed. | | |

## Appendix 2: Checking the balancing of important variables across the treatment arms

| SI Table 2. Baseline summary statistics and randomization tests | | | | | | | | | | | | | | | |
| --- | --- | --- | --- | --- | --- | --- | --- | --- | --- | --- | --- | --- | --- | --- | --- |
|  | Cluster A |  | Cluster B |  | Control |  | *p*-value |  | *p*-value |  | *p*-value |  | *p*-value |  | Observations |
|  | (1) |  | (2) |  | (3) |  | ∆ [(1), (2)] |  | ∆ [(1), (3)] |  | ∆ [(2), (3)] |  | (all) |  | (8) |
| *Respondent Attributes* |  |  |  |  |  |  |  |  |  |  |  |  |  |  |  |
| Baseline age | 35.52 |  | 36.37 |  | 36.33 |  | 0.24 |  | 0.53 |  | 0.97 |  | 0.49 |  | 846 |
|  | (0.65) |  | (0.23) |  | (1.09) |  |  |  |  |  |  |  |  |  |  |
| Illiterate (1 = yes) | 0.63 |  | 0.67 |  | 0.63 |  | 0.49 |  | 0.95 |  | 0.46 |  | 0.72 |  | 846 |
|  | (0.03) |  | (0.04) |  | (0.03) |  |  |  |  |  |  |  |  |  |  |
| Has 1-7 years of schooling (1 = yes) | 0.14 |  | 0.11 |  | 0.11 |  | 0.37 |  | 0.13 |  | 0.96 |  | 0.24 |  | 846 |
|  | (0.01) |  | (0.03) |  | (0.01) |  |  |  |  |  |  |  |  |  |  |
| Married (1 = yes) | 0.89 |  | 0.89 |  | 0.91 |  | 0.89 |  | 0.39 |  | 0.36 |  | 0.60 |  | 846 |
|  | (0.01) |  | (0.02) |  | (0.02) |  |  |  |  |  |  |  |  |  |  |
|  |  |  |  |  |  |  |  |  |  |  |  |  |  |  |  |
| *Household Characteristics* |  |  |  |  |  |  |  |  |  |  |  |  |  |  |  |
| Household size | 6.32 |  | 6.51 |  | 6.45 |  | 0.17 |  | 0.56 |  | 0.77 |  | 0.38 |  | 846 |
|  | (0.1) |  | (0.09) |  | (0.19) |  |  |  |  |  |  |  |  |  |  |
| Head is male (1 = yes) | 0.95 |  | 0.96 |  | 0.96 |  | 0.88 |  | 0.80 |  | 0.87 |  | 0.97 |  | 846 |
|  | (0.02) |  | (0.01) |  | (0.01) |  |  |  |  |  |  |  |  |  |  |
| Head's age | 43.66 |  | 45.40 |  | 43.73 |  | 0.17 |  | 0.95 |  | 0.31 |  | 0.37 |  | 846 |
|  | (0.28) |  | (1.16) |  | (1.07) |  |  |  |  |  |  |  |  |  |  |
| Head is married (1 = yes) | 0.94 |  | 0.94 |  | 0.94 |  | 0.95 |  | 0.72 |  | 0.90 |  | 0.94 |  | 846 |
|  | (0.01) |  | (0.03) |  | (0.01) |  |  |  |  |  |  |  |  |  |  |
| Head's caste: OBC (1 = yes) | 0.73 |  | 0.69 |  | 0.68 |  | 0.71 |  | 0.73 |  | 0.95 |  | 0.91 |  | 846 |
|  | (0.09) |  | (0.08) |  | (0.13) |  |  |  |  |  |  |  |  |  |  |
| Head's caste: SC (1 = yes) | 0.19 |  | 0.25 |  | 0.26 |  | 0.52 |  | 0.56 |  | 0.94 |  | 0.76 |  | 846 |
|  | (0.07) |  | (0.07) |  | (0.10) |  |  |  |  |  |  |  |  |  |  |
| Head's caste: ST (1 = yes) | 0.07 |  | 0.06 |  | 0.06 |  | 0.75 |  | 0.83 |  | 0.92 |  | 0.95 |  | 846 |
|  | (0.03) |  | (0.02) |  | (0.03) |  |  |  |  |  |  |  |  |  |  |
| Head is illiterate (1 = yes) | 0.27 |  | 0.24 |  | 0.23 |  | 0.46 |  | 0.29 |  | 0.90 |  | 0.54 |  | 846 |
|  | (0.03) |  | (0.03) |  | (0.02) |  |  |  |  |  |  |  |  |  |  |
| Head has 1-7 years of schooling (1 = yes) | 0.15 |  | 0.17 |  | 0.14 |  | 0.49 |  | 0.46 |  | 0.24 |  | 0.46 |  | 846 |
|  | (0.01) |  | (0.02) |  | (0.01) |  |  |  |  |  |  |  |  |  |  |
|  |  |  |  |  |  |  |  |  |  |  |  |  |  |  |  |
| *Socio-Economic Status* |  |  |  |  |  |  |  |  |  |  |  |  |  |  |  |
| Household has a toilet? (1 = yes) | 0.02 |  | 0.06 |  | 0.10 |  | 0.15 |  | 0.18 |  | 0.48 |  | 0.17 |  | 846 |
|  | (0.01) |  | (0.02) |  | (0.06) |  |  |  |  |  |  |  |  |  |  |
| Assets Index | 4.98 |  | 5.30 |  | 5.31 |  | 0.38 |  | 0.34 |  | 0.97 |  | 0.62 |  | 846 |
|  | (0.32) |  | (0.16) |  | (0.10) |  |  |  |  |  |  |  |  |  |  |
|  |  |  |  |  |  |  |  |  |  |  |  |  |  |  |  |
| *Hygiene and Health* |  |  |  |  |  |  |  |  |  |  |  |  |  |  |  |
| Open defecation by the respondent (self-reported) (1 = yes) | 0.98 |  | 0.95 |  | 0.91 |  | 0.15 |  | 0.23 |  | 0.48 |  | 0.20 |  | 846 |
|  | (0.01) |  | (0.02) |  | (0.06) |  |  |  |  |  |  |  |  |  |  |
| Open defecation by the respondent (enumerator observation) (1 = yes) | 0.98 |  | 0.94 |  | 0.90 |  | 0.15 |  | 0.18 |  | 0.48 |  | 0.17 |  | 846 |
|  | (0.01) |  | (0.02) |  | (0.06) |  |  |  |  |  |  |  |  |  |  |
| Frequency of open defecation/using toilets | 2.04 |  | 2.09 |  | 2.18 |  | 0.32 |  | 0.27 |  | 0.48 |  | 0.34 |  | 846 |
|  | (0.02) |  | (0.04) |  | (0.12) |  |  |  |  |  |  |  |  |  |  |
| Handwash with soap after defecation (1 = yes) | 0.09 |  | 0.10 |  | 0.13 |  | 0.95 |  | 0.35 |  | 0.44 |  | 0.63 |  | 846 |
|  | (0.01) |  | (0.02) |  | (0.04) |  |  |  |  |  |  |  |  |  |  |
| Soap observed at the handwashing station (1 = yes) | 0.36 |  | 0.40 |  | 0.44 |  | 0.59 |  | 0.04 |  | 0.58 |  | 0.10 |  | 846 |
|  | (0.03) |  | (0.07) |  | (0.02) |  |  |  |  |  |  |  |  |  |  |
|  |  |  |  |  |  |  |  |  |  |  |  |  |  |  |  |
| *Mother and Child Characteristics* |  |  |  |  |  |  |  |  |  |  |  |  |  |  |  |
| Mother's BMI | 21.38 |  | 21.91 |  | 21.42 |  | 0.50 |  | 0.96 |  | 0.61 |  | 0.78 |  | 213 |
|  | (0.35) |  | (0.67) |  | (0.66) |  |  |  |  |  |  |  |  |  |  |
| Child breastfed exclusively for the first 6 months (1 = yes) | 0.99 |  | 0.98 |  | 1.00 |  | 0.71 |  | 0.26 |  | 0.31 |  | 0.31 |  | 213 |
|  | (0.009) |  | (0.02) |  | (0.00) |  |  |  |  |  |  |  |  |  |  |
| Child is female (1 = yes) | 0.53 |  | 0.42 |  | 0.52 |  | 0.20 |  | 0.91 |  | 0.26 |  | 0.42 |  | 213 |
|  | (0.04) |  | (0.08) |  | (0.05) |  |  |  |  |  |  |  |  |  |  |
| Child suffered from diarrhea in the past one month (1 = yes) | 0.25 |  | 0.25 |  | 0.31 |  | 0.98 |  | 0.66 |  | 0.54 |  | 0.82 |  | 213 |
|  | (0.10) |  | (0.03) |  | (0.10) |  |  |  |  |  |  |  |  |  |  |
| Height-for-age z-scores for children under 5 | -0.86 |  | -0.99 |  | -0.88 |  | 0.60 |  | 0.96 |  | 0.77 |  | 0.86 |  | 213 |
|  | (0.16) |  | (0.18) |  | (0.32) |  |  |  |  |  |  |  |  |  |  |
| Weight-for-age z-scores for children under 5 | -1.37 |  | -1.54 |  | -1.50 |  | 0.48 |  | 0.56 |  | 0.88 |  | 0.73 |  | 213 |
|  | (0.15) |  | (0.18) |  | (0.17) |  |  |  |  |  |  |  |  |  |  |
| Weight-for-height z-scores for children under 5 | -1.26 |  | -1.46 |  | -1.42 |  | 0.39 |  | 0.39 |  | 0.87 | \|  \| \| --- \| | \| 0.60. \| \| --- \| |  | 213 |
|  | (0.14) |  | (0.18) |  | (0.12) |  |  |  |  |  |  |  |  |  |  |
| Levels of *E.coli* in drinking water (safe, unsafe) | Safe |  | Safe |  | Safe |  |  |  |  |  |  |  |  |  |  |
| Notes: Columns (1)-(3) depict the experimental arm specific means and the corresponding standard errors are reported in parenthesis below the coefficient. The standard errors are clustered at the village level. * p < 0.10, ** p < 0.05, *** p < 0.01. | | | | | | | | | | | | | | | |

## Appendix 3: Detailed analysis of toilet construction and adoption outcomes

| SI Table 3: Impact of treatments on toilet ownership and open defecation | | | | | |
| --- | --- | --- | --- | --- | --- |
|  | Toilet ownership | |  | Open defecation | |
|  | (1) | (2) |  | (3) | (4) |
| Importance of BCC + toilet construction (cluster A) | 0.92**** | 0.92**** |  | -0.93**** | -0.93**** |
|  | (0.05) | (0.05) |  | (0.03) | (0.03) |
| *(Cluster-Robust p-Value)* | 0.000 | 0.000 |  | 0.000 | 0.000 |
| *(Wild Bootstrap p-Value)* | 0.008 | 0.000 |  | 0.002 | 0.000 |
| Importance of toilet construction (cluster B) | 0.77**** | 0.77**** |  | -0.43**** | -0.43**** |
|  | (0.06) | (0.06) |  | (0.02) | (0.02) |
| *(Cluster-Robust p-Value)* | 0.000 | 0.000 |  | 0.000 | 0.000 |
| *(Wild Bootstrap p-Value)* | 0.000 | 0.008 |  | 0.000 | 0.000 |
|  |  |  |  |  |  |
|  |  |  |  |  |  |
| Individual fixed effects |  |  |  | Yes | Yes |
| Household fixed effects | Yes | Yes |  |  |  |
| Survey fixed effects | Yes | Yes |  | Yes | Yes |
| Village fixed effects |  | Yes |  |  | Yes |
| Unit of analysis | Household level | Household level |  | Respondent level | Respondent level |
| Observations | 1692 | 1692 |  | 1692 | 1692 |
| Villages | 15 | 15 |  | 15 | 15 |
| *Notes*: Standard errors are reported in parenthesis below the coefficient and are clustered at the village-level. Asterisk denotes significance. † p < 0.15, * p < 0.10, ** p < 0.05, *** p < 0.01, **** p < 0.001. | | | | | |

Columns (1) to (4) of SI Table 3 report the difference-in-difference OLS estimates for toilet coverage and open defecation. For this analysis, we only use the households present in both surveys. The DID estimates report that the increase in toilet ownership in cluster A was about 92 percentage points, while for cluster B, it was 77 percentage points (when compared to the control group). The estimates also indicate a corresponding decrease of 93 percentage points and 43 percentage points in open defecation of the respondents in clusters A and B, respectively, versus the control group. Keep in mind that for cluster A, the DID estimates suggest that the decline in open defecation is more than the toilets built relative to the control group because when using the panel data for analysis, open defecation increased slightly in the control group in the endline. Adding village fixed effects in columns (2) and (4) does not change our results.

To gain more insights into understanding the open defecation patterns among different clusters, genders, and across seasons, our study tracks the monthly toilet usage of the heads of the households and their spouses, from all the households participating in this experiment (see Fig 1, Appendix 3).

 **

Fig 1: The graphs depict the monthly toilet usage of the index man and woman of the household. All responses are self-reported.

Note that the OD in cluster A was considerably reduced for male and female respondents by January 2018 as they had been using *kuchha* (temporary) toilets for the past four to five months.

Three clear patterns emerge from these graphs:

1. There is a discernible difference in the toilet use patterns of the two treatment arms, indicating the key contribution of behavior change communication.

The data strongly underscores the necessity of integrating behavioral change initiatives with toilet construction to achieve rapid and sustained reductions in open defecation practices,

1. Women’s open defecation is lower than that of the men in cluster B.

This could indicate that women are more responsive to behavioral change campaigns or have stronger motivations to shift away from open defecation, due to privacy and safety concerns.

1. The seasonal variation in cluster B is highlighted during the months of monsoon (June to August). The tendency to not OD and use toilets did not transition into a habit as the respondents resumed their pre-monsoon defecation patterns after August 2018, yet again emphasizing the significance of behavior change communication (complementing the construction of toilets) to eradicate OD from the community.
2. The comparison of enumerator observations and self-reported rates of open defecation among women reveals a remarkable consistency in the data across the three clusters, suggesting high reliability in the reporting of behavior change. This comparability reinforces the validity of using self-reported data in similar studies, especially when coupled with observational verification.
