# Supplementary material for: Addressing information and credit barriers to making India open defecation free and improving child health: Evidence from a cluster randomized trial in rural India
Source: PLoS One. 2025 Feb 27;20(2):e0318198. doi: 10.1371/journal.pone.0318198 (PMC11867337; doi:10.1371/journal.pone.0318198)
Supplement: Attachment — Submitted filename: 1. Response to Reviewers.docx [file pone.0318198.s002.docx]

Reviewer #1: Thank you for the opportunity to review this article. It is written on a very important topic and can potentially provide very important contributions to the field.

Unfortunately, I cannot recommend it for publication at this stage. Here are my suggestions to improve the manunscript:

1. The literature review has to be expanded. You are presenting current efforts to improve sanitation in India as only focused on the supply side of the problem. This is false. In fact, SBM had a big demand component, which is completely overlooked in the paper. When you refer to limited implementation of behavioral change, you need to be more specific about how your intervention is different from what was proposed/implemented before.

Thank you for your feedback regarding the literature review. We understand that this is a gap which needed to be addressed properly. We have addressed this concern by adding a section that discusses all the barriers associated with the limited adoption of toilets in India, using the studies that did impact the evaluation of TSC and SBM campaigns. We also discuss the demand-side components of the SBM and how studies have documented that despite the success of SBM in enhancing access to toilets, its behaviour change either did not reach the beneficiaries or was coercive in nature. This is added from page 3 onwards of the document titled “Revised manuscript” or page 4 onwards of the document titled “Revised Manuscript with tracked changes”.

The same section discusses the contribution of our research design and how it builds on the limitations of the previous studies in detail. For instance, discussing points in brief- First, our intervention differentiates itself by specifically addressing the limitations observed in prior behavioral change strategies, such as the reliance on coercive methods or SBM reaching only limited beneficiaries (see page 6). Our research design focuses on the (1) rigorous implementation of all the activities of CLTS (as is shown in Appendix A) and (2) is disseminated to *all* the people in the villages. This is a more community-driven approach that focuses on intrinsic motivation and long-term sustainability.

Second, our intervention also differs from previously implemented programs. Unlike previous national sanitation campaigns that were heavily focussed on building toilets (Dickinson et al., 2015; Hammer & Spears, 2016; Patil et al., 2014) and found limited impact on adoption of toilets and child health improvement, our study includes a behavior change component. And unlike experiments in developing countries that implemented only CLTS-like behavior change programs and found that standalone CLTS intervention is not effective for toilet adoption (Cameron et al., 2019; Guiteras et al., 2015), we included both CLTS along with subsidised toilet construction. Hence our study is different from the previous studies as it is emphasising the joint efficacy of *both* *universal* behavior change (removal of information constraint) and building subsidised toilet construction (removal of credit constraint) for *every* household that is opting for the subsidy. The interventions, unlike previous studies, are provided to *all* the households in one of the intervention arms.

We have added an entire section on “Research Design and its Contribution” from page 6 of the document titled “Revised manuscript” or “Revised Manuscript with tracked changes”. This delineates all the contributions that our study is making when compared to previous interventions.

2. The paragraph on CLTS makes it seem like CLTS has never before been implemented in India. In fact, we know that many CLTS attempts failed (see for example Leong 2020, "Narratives of sanitation"). If your approach is merely CLTS, why is it different this time?

We acknowledge the reviewer’s observation regarding the mixed outcomes of previous Community-Led Total Sanitation (CLTS) interventions in India. Leong (2020) highlights that traditional CLTS methods, which rely heavily on invoking shame and disgust to motivate behavior change, often fail to produce sustainable results. This failure can be attributed to their coercive nature and a lack of cultural sensitivity, as well as their inability to address the deeply entrenched socio-cultural norms and environmental identities tied to sanitation practices.

Our approach builds on these lessons by addressing key limitations of earlier CLTS implementations:

1. **Cultural Sensitivity and Positive Reinforcement**:
   Unlike traditional CLTS methods, which often alienate communities by focusing on negative emotions, our intervention emphasizes intrinsic motivation and positive messaging. We align sanitation practices with values such as dignity, health, and modernity, ensuring that behavior change resonates with local aspirations and identities. This is particularly important given the findings from Leong (2020), which demonstrate that cultural norms and environmental identities—such as preferences for open spaces (a “green identity”)—play a significant role in shaping sanitation behaviors.

You can see the details of the activities deployed under CLTS in this project and its successful dissemination in Table A1, Appendix 1. The table shows how nearly all the members of the community were present during all the activities of CLTS

| **Table A1.** Intervention Check | | |
| --- | --- | --- |
|  | Sample Mean | Observations |
|  | (1) | (2) |
| % of respondents participated in following BCC activities (in cluster A) |  |  |
| Fecal calculation | 96.76 | 328 |
| OD mapping | 97.64 | 331 |
| Transect walk | 96.76 | 328 |
| Calculating health expenses | 97.94 | 332 |
| ODF pledge/commitment | 88.50 | 300 |
| Handwashing training | 96.46 | 327 |
| Toilet maintenance training | 88.20 | 299 |
| Natural leader selection | 74.34 | 252 |
| Action plan | 75.81 | 257 |
| Fecal-oral demonstration | 97.94 | 332 |
| Follow-up monitoring | 89.97 | 305 |
| Arouse dignity (eating other's feces) | 97.64 | 331 |
| CLTS committee selection | 58.70 | 199 |

2. **Integration of Behavior Change with Infrastructure Support**:
   Previous CLTS campaigns often operated independently of toilet construction, leaving motivated households without the means to adopt improved sanitation practices. In contrast, our intervention combines universal subsidized individual household toilet construction with a community-led behavior change campaign. This integrated approach addresses both supply-side constraints (through subsidies) and demand-side barriers (through BCC), ensuring households have both the means and the motivation to adopt and sustain toilet use.
3. **Universal Outreach and Inclusivity**:
   Traditional CLTS campaigns have often targeted specific groups within communities, leading to fragmented outcomes and limited behavioral diffusion. Our approach ensures *universal* coverage, engaging every household in the intervention clusters. This not only fosters collective accountability but also helps normalize toilet usage as a community-wide practice, reducing resistance from those who might otherwise continue open defecation. (See table A1, Appendix A1).
4. **Focus on Sustainability**:
   As noted in Leong’s work, the effectiveness of shame-based tactics diminishes over time, and behavioral changes are often not "sticky." Our approach prioritizes long-term sustainability by fostering community ownership of sanitation outcomes and leveraging social norms to embed these changes into daily practices. This aligns with recommendations from the paper, which advocate bridging the gap between "green" and "technology" identities by framing toilet use as both environmentally and socially desirable.

In summary, our intervention represents an evolution of the CLTS framework, addressing the structural and cultural challenges that have hindered its success in the past. By integrating behavior change communication with universal access to subsidized toilets and focusing on positive, inclusive messaging, our approach seeks to achieve sustainable sanitation outcomes and foster an open defecation-free environment.

3. After reading the paper I am not confident I understand exactly what the intervention included. You need to add a section describing the intervention in more detail.

That is a great point. We have added an entire section on “Research Design and its Contribution” from page 6 of the document titled “Revised manuscript” or “Revised Manuscript with tracked changes”. We hope that this makes it easier for the readers to comprehend the mature of interventions. Besides adding the details of the interventions with more clarity, we have also added Table 1, which can make it easier for the readers to refer to the interventions.

4. You chose a district with a 17% coverage rate. How is it that the coverage is so much lower in your sample (5.32%)?

Thank you for your insightful observation. While the district has an overall coverage rate of 17%, it is important to note that this figure reflects a broad average across various town and villages in the district, some of which may have higher coverage rates than others. Given that our study specifically focuses on rural villages within the district, where access to resources and infrastructure may be more limited, it is expected that the coverage rate in these areas would be lower. Consequently, the sample from these rural villages reflects a lower toilet coverage of 5.32%, which is consistent with the challenges faced in these regions.

5. How was the sample size determined? Why did you only choose 5 villages per cluster?

The sample size was determined using a cluster-based sampling approach, where each cluster consisted of 5 villages. In each of these villages, we included *every* household within the village, with a total of 866 households for 15 villages. This approach ensured comprehensive coverage and a highly representative sample, as all households in each village were included, rather than just a random selection.

The decision to select 5 villages per cluster was primarily driven by budget constraints, which limited the number of villages we could incorporate while maintaining the statistical integrity of the study.

To ensure the chosen sample would still yield reliable results, we ran power calculations to assess whether the impact of the intervention could be detected at child health level, i.e. if the sample was powered enough to detect the impact on children’s WAZ scores. These calculations confirmed that the sample size, based on the full census of households, would be sufficient to observe the intended impacts.

6. While I am not a great supporter of balance tests in studies, it is very important in a study with that small sample size. Why was the coverage in cluster A so much lower than in cluster B and control? 2% vs 10% is a massive difference. You further report sample sizes as 846 when coverage is a village-level variable so your sample can't be greater than 15. You need to motivate those differences in coverage.

Thank you for highlighting the importance of balance tests, especially in studies with smaller sample sizes. We acknowledge your observation regarding the coverage differences between cluster A (2%) and control group (10%). While this difference may seem substantial at first glance, it is important to note that statistical analysis during the balancing test (table A2, Appendix 2) showed that this variation was not statistically significant, indicating that it is unlikely to have affected the overall findings of our study.

The figure of 866 (attrition of 20 households in endline = 846 households) represents the total number of households included in our study across the 15 villages, based on a complete census. That is, *every* household in *all* the villages received the interventions and are a part of baseline and endline surveys. So, the study sample size of 866 households is inclusive of all the households in the 15 villages. That is, no random sampling or selection of sampled households was done as the interventions and surveys occurred at the census level.

The coverage percentages we reported refer to the proportion of these households that had built individual household toilets, provides insight into the impact at the household level within each cluster. When we mention sanitation coverage with respect to 846 households, it is the same as coverage across 15 villages.

We have clarified this on page 7 of the document titled “Revised manuscript” or “Revised Manuscript with tracked changes”.

**All 866 households across the 15 villages participated in the study, representing a comprehensive *census* of the entire population in these villages.**

7. I am very confused about your choice of a dependent variable. The title doesn't suggest anything about using health outcomes and makes it seem like you were testing every possible dependent variable to find any effect.

Thank you for your feedback regarding the clarity of our choice of the dependent variable. Our primary objective was to examine the impact of alleviating both information and credit constraints on reducing OD and improving child health outcomes in rural India.

We understand that it did not come across like that and we have made minor changes in the abstract and introduction to give due importance to the child health outcomes.

Most importantly, we have added a subsection titled “Mechanism Linking Improved Sanitation and Child Health” that highlights the literature from biomedical sciences to understand the pathways through which water, sanitation, and hygiene interventions can lead to improved health. This will strengthen the rationale as to why our paper is also focussing on child health outcomes. You can find this section from page 5 of the document titled “Revised manuscript” and “Revised Manuscript with tracked changes”.

Taking your suggestion into account, we have revised the title to: **Addressing Information and Credit Barriers to Making India Open Defecation Free and Improving Child Health: Evidence from a Cluster Randomized Trial in Rural India**

8. You mention monthly follow-ups. Why are they not included in the identification strategy? Why is only the endline included? What was the purpose of monthly follow-ups?

We appreciate your question regarding the role of monthly follow-ups and their exclusion from the identification strategy.

The primary purpose of the monthly surveys conducted from February 2018 to December 2018 was to track trends in toilet adoption over time. These surveys focused only on collecting self-reported toilet usage data from household heads and primary female respondents, along with enumerator observations for verification. The aim was to monitor short-term variations in adoption behavior across interventions, genders, and seasons, providing a more granular understanding of behavioral shifts. However, these monthly follow-ups were not comprehensive surveys; they did not include the broader socioeconomic and hygiene-related modules that were part of the baseline and endline surveys. Detailed results from monthly surveys are discussed in Appendix A3.

We have clarified this on page 9 of the document titled “Revised manuscript” or page 10 of “Revised Manuscript with tracked changes”.

***From February 2018 to December 2018, we conducted monthly surveys with households to track toilet adoption trends. These surveys involved interviewing both the household heads and the primary female respondents only regarding their toilet adoption. Additionally, enumerators verified toilet usage during these visits. This approach allowed us to capture variations in toilet adoption across different interventions, genders, and seasonal patterns, providing a comprehensive understanding of the trends over time. We did not ask about other modules from the baseline survey in these monthly surveys.***

9. WAC is a very misleading variable. Is it plausible to fix malnutrition outcomes in 2 years? Children also aged in that period so you are comparing children at different ages in the baseline and endline. If OD disproportionately affects infants, you might see a correction due to aging /mortality. After 2 years of the study, how do you include new-born children who didn't have data at the baseline?

We understand your concern and while all the malnutrition outcomes like height-for-age cannot be fixed in the short period of 2 years, outcomes related to weight can be changed in a relatively shorter period of time as weights are quick to respond to short-term changes in the quantity and quality of diets and changes in sanitation environment, unlike outcomes like height-for-age (Arndt et al., 2016). Similarly, (Dickinson et al., 2015) who also conducted an evaluation of a sanitation campaign in India, found an increase in the ownership of toilets from 7% to 35% which led to a 0.26-0.31 increase in children’s WAZ scores, *after one year of implementation of the program*.

Yes, that is rightly pointed out. Children will be aged differently at the baseline and endline and we are comparing the same set of children, but since the weight-for-age z-score is *adjusted for age by the World Health Organisation* (WHO), it should allay this concern. We only included 213 children who were surveyed both in the baseline and endline. Hence, the newborns surveyed for the first time in the endline were not a part of the data analysis.

Additionally, we would like to emphasize that our analysis compares changes in the intervention clusters against control clusters where no sanitation interventions were implemented. This comparative approach accounts for both the age effect and the sanitation effect, allowing us to isolate the potential impact of the intervention on the WAZ scores.

10. What was the mortality/attrition in the sample?

There was attrition of 20 households in the endline. No attrition for children.

We have clarified this on page 9 of the document titled “Revised manuscript” or page 11 of “Revised Manuscript with tracked changes”.

***Of the 866 households covered in the baseline survey, 846 (97.6 percent) participated in the endline survey. Of the 846 households, 339 (40%) belonged to cluster A, 270 (32%) belonged to cluster B, and 237 (28%) in the control arm. Of the 278 children who were surveyed in the baseline, only 213 children who were less than five years of age in the baseline and endline were included for analysis. A panel data or the same set of 1692 households (846 households in the baseline and endline) and the same set of 426 children (213 children in the baseline and endline) are used for all the following analyses.***

11. Have you considered desirability effects in responses? You mention yourself that observations didn't confirm hand-washing practices in a large number of cases but dismiss the finding. Is it possible that respondents over-report toilet use to make your surveyors happy?

Thank you for your insightful comment regarding the potential for social desirability bias in our respondents' answers. We agree that this is a valid concern, as respondents may indeed over-report positive behaviors, such as using toilets, to align with perceived social expectations or to please the surveyors.

To mitigate this, we implemented several measures during the data collection process.

First, we trained surveyors to emphasize that responses would remain confidential and that there were no right or wrong answers. This approach was intended to reduce the pressure on respondents to provide socially desirable responses.

Second, while it is true that direct observation of hand-washing was limited in certain cases, we did not dismiss this finding. This is what we had mentioned in terms of handwashing- While 93% of people are self-reporting higher washing of hands with soap in cluster A (compared to the control group), the enumerators found that only 62% had soaps (see page 13 of document titled “Revised manuscript” or page 16 of Revised Manuscript with tracked changes”). The difference could be explained as people prefer washing their hands with a powder detergent after toilet use than soaps, and the enumerators had observed only soaps. Rather, we considered this finding as part of the broader context of behavior verification, which highlights the challenges of accurately capturing self-reported behaviors. Leaving aside washing hands with powdered detergent, we know for sure that compared to the control group, 62% of respondents are washing their hands with soaps, which in itself is an improvement.

Third, we incorporated objective measures such as child health outcomes into our analysis which provides additional validation for our findings. Improvements in child health, such as reductions in diarrhea prevalence and increases in weight-for-age z-scores, serve as objective indicators that align with increased toilet use. These health outcomes are less prone to desirability bias and strengthen our confidence that reported behavioral changes (increase in toilet use) are genuinely associated with tangible benefits. The consistency between self-reported toilet use and positive child health outcomes reinforces the reliability of our data and mitigates concerns about over-reporting, as is usually the case in the studies within the sanitation-child health nexus.

Fourth, and most importantly, we have enumerator verification of toilet monthly usage (as is shown in Appendix A3), which is similar to the self-reported toilet use by female respondents. We have added an additional point to highlight the consistency between self reported and enumerator verified observations on page 26 of the document titled “Revised manuscript” or page 27 of “Revised Manuscript with tracked changes”.

***The comparison of enumerator observations and self-reported rates of open defecation among women reveals a remarkable consistency in the data across the three clusters, suggesting high reliability in the reporting of behavior change. This comparability reinforces the validity of using self-reported data in similar studies, especially when coupled with observational verification.***

Finally, like most RCT studies on sanitation, we acknowledge that despite these precautions, some bias may still exist. This is why we have mentioned this concern in the limitations on page 17 of the document titled “Revised manuscript” or page 19 of “Revised Manuscript with tracked changes”.

***Next, respondents’ self-reported use of toilets might be subject to bias*** (Clasen et al., 2012; Jenkins et al., 2014)***. To mitigate this issue, we include child health outcomes in our analysis.***

12. The most important thing I want you to focus on is positioning your findings in the body of literature. CLTS has failed many times before in India. How is your intervention so successful while nothing else including SBM worked? If you indeed discovered a holy grail of OD in India, I have nothing but respect for you, but given the quality of the evidence I suspect your long-term efficacy will be modest at best.
If you indeed find your intervention to have an almost universal success, you need to make sure the recipe is publicly available and make the intervention details available to the readers.

Thank you for your comment. This is a useful observation that will enrich our paper and help the readers understand how our experiment was able to combat OD in India.

To address your concern, we have added one section from page 3 of the document titled “Revised manuscript” or page 4 of Revised Manuscript with tracked changes”. This section discusses the barriers to the adoption of toilets in the Indian context.

Next, we elaborate on how understanding these barriers to toilet usage in India through a rigorous literature review led to the development of our research design that incorporates these limitations and the contribution of this research design. You can find this on page 6 of the document titled “Revised manuscript” or page 6 of “Revised Manuscript with tracked changes”.

Reviewer #2:

1. When using acronyms, please provide full form when it is used in the first place. For example, WAZ in the abstract.

Thank you for your comments. This is a good observation, have edited it in the document.

2. There is confusion in the timeline-related description. On page no. 12, in the second paragraph authors have written “conducting a monthly survey… from February 2018 to December 2019.” And then, in the next paragraph, they wrote “The final endline survey was carried out in December 2018 and January 2019.” The confusion is why the endline survey was conducted in the middle of the monthly survey. And how these two types of surveys are different from each other?

That is also a good observation. The survey from February 2018 to December 2018 was a monthly survey about toilet usage and its results are discussed in Appendix 3, Figure A3. December 2019 was a typo and we have corrected it.

3. Another confusion is you mentioned 846 households participated in the endline survey; then how exactly is the double number (1692) of households included in the analyses? Also, you reported 278 children below five years of age at one place while at the other place, you mentioned 426 children and again at a place, you wrote 213 children.

Another good point. We had two observations for each household and child in the baseline and endline as this is a panel data. We have clarified it in the following way on page 9 of document titled “Revised manuscript” or page 11 of Revised Manuscript with tracked changes”-

***Of the 866 households covered in the baseline survey, 846 (97.6 percent) participated in the endline survey. Of the 846 households, 339 (40%) belonged to cluster A, 270 (32%) belonged to cluster B, and 237 (28%) in the control arm. Of the 278 children who were surveyed in the baseline, only 213 children who were less than five years of age in the baseline and endline were included for analysis. A panel data or the same set of 1692 households (846 households in the baseline and endline) and the same set of 426 children (213 children in the baseline and endline) are used for all the following analyses.***
